# Effects of hamstring conditioning contraction on landing biomechanics and surface EMG: a randomized controlled trial

**DOI:** 10.3389/fbioe.2026.1702762

**Published:** 2026-06-12

**Authors:** Tiancheng Yu, Bocheng Chen, Ziyan Ye, Jiaxin Wu, Jingyuan Jiang, Guoxiang Wang

**Affiliations:** 1 School of Physical Education, Nanjing Tech University, Nanjing, China; 2 Physical Education and Sports School of Soochow University, East Campus of Soochow University, Suzhou, Jiangsu, China

**Keywords:** hamstring activation, landing biomechanics, non-contact ACL injury risk, post-activation potentiation, wavelet decomposition

## Abstract

**Objective:**

This study aimed to investigate the effects of post-activation potentiation (PAP) of the hamstrings on surface electromyography (sEMG) characteristics and knee joint biomechanics during landing.

**Methods:**

This two-arm, parallel-group randomized controlled trial (1:1 allocation) included 34 male collegiate athletes assigned to a PAP group (n = 17) or a control group (n = 17). The PAP group performed a 5-s hamstring MVIC followed by an 8-min interval, whereas the control group remained seated quietly for an equivalent 8-min interval without conditioning contraction. Drop-landing tasks were completed before and after the intervention. Kinematic, kinetic, and sEMG data were collected synchronously. Wavelet decomposition was applied to hamstring sEMG signals across four frequency bands. Primary outcomes were wavelet-derived EMG power in the 60–200 Hz frequency band for the biceps femoris and semitendinosus/semimembranosus during landing; all other EMG bands, biomechanical variables, iEMG, and co-contraction indices were considered secondary/exploratory. Trial registration: Not registered.

**Results:**

For the primary outcomes, the PAP group showed significant pre–post increases in wavelet-derived EMG power within the 60–200 Hz frequency band for both the semitendinosus/semimembranosus and biceps femoris, whereas no significant pre–post changes were observed in the control group. Post-intervention between-group comparisons further showed significantly higher 60–200 Hz wavelet power in the PAP group than in the control group for the semitendinosus/semimembranosus (FDR-adjusted p = 0.014, 95% CI: 2100.466–6465.455) and biceps femoris (FDR-adjusted p = 0.045, 95% CI: 605.924–1131.846). Among secondary and exploratory outcomes, frequency-domain changes were observed in other bands, including increased 200–480 Hz wavelet power in both muscles and decreased 0–20 Hz wavelet power in the biceps femoris. Biomechanically, the PAP group demonstrated greater maximal knee flexion angles (p < 0.001, 95% CI for PAP − Control [6.374, 10.699]) and reduced sagittal- (p = 0.043, 95% CI for PAP − Control [−0.323, −0.010]) and transverse-plane (p = 0.002, 95% CI for PAP − Control [−0.360, −0.068]) knee joint moments compared with the control group, indicating altered joint loading patterns during landing.

**Conclusion:**

Hamstring PAP was associated with acute changes in hamstring surface EMG characteristics a nd landing biomechanics, including increased 60–200 Hz wavelet-derived EMG power, greater knee flexion, and lower frontal- and transverse-plane knee joint moments.

## 1 Introduction

High-altitude landing is a common action in many sports and is one of the highest-risk movements for non-contact anterior cruciate ligament (ACL) injuries (Arundale et al., 2022). Non-contact ACL injuries occur when the injury happens without direct external contact and are often closely associated with the activation patterns of the muscles around the knee joint (Owoeye et al., 2020). Research has shown that when the quadriceps are overly activated relative to the hamstrings during landing, the knee experiences higher knee extension moments and anterior shear forces, significantly increasing the load on the ACL (Lopes et al., 2018; Dewig et al., 2020). Therefore, training to strengthen the hamstrings is often used to reduce the risk of non-contact ACL injuries. However, such training typically requires long periods, consuming considerable time and energy. Finding ways to rapidly enhance hamstring strength to reduce injury risk before completing full injury prevention training remains an important area for further study.

Post-activation potentiation (PAP) is a physiological phenomenon where muscle strength and contraction rate significantly increase shortly after maximal or near-maximal resistance exercises (Abbate et al., 2000; Gilbert and Lees, 2005). Studies indicate that PAP can be induced by dynamic or isometric contractions, with Maximum Voluntary Isometric Contraction (MVIC) being particularly effective in eliciting PAP (Zero and Rice, 2021). Skurvydas et al. (2019) found that a 5-s MVIC can significantly induce PAP. Regarding the timing of the peak PAP effect, Kilduff et al. (2008) suggested an optimal recovery time of 8–12 min, while Lowery et al. (2012) reported 4–8 min as optimal for high-level athletes.

PAP has been widely applied to enhance athletic performance, particularly in explosive movements such as sprinting and jumping. For example, it has been used to improve sprinting performance in athletes (Yetter and Moir, 2008; Okuno et al., 2013) and increase jump height (Mitchell and Sale, 2011). However, although PAP has been discussed in the context of sports injury prevention, its specific role remains unclear, particularly regarding whether enhanced hamstring function can acutely modify neuromuscular activation and knee joint biomechanical characteristics during landing (Ullman et al., 2021).

It is important to note that the hamstrings consist of different muscles, including the biceps femoris and semitendinosus/semimembranosus, which have distinct anatomical and functional roles. The biceps femoris primarily controls knee joint external rotation, while the semitendinosus/semimembranosus play key roles in knee stability and internal rotation (McCann and Flanagan, 2010). However, knee stability during landing should not be interpreted solely as the isolated function of individual hamstring muscles. From the perspective of muscle synergy, the central nervous system organizes multiple muscles into functional modules to simplify the control of complex multi-joint movements (d’Avella and Bizzi, 2005; Ting and McKay, 2007). During landing, the hamstrings and quadriceps work cooperatively within a broader neuromuscular control strategy. The quadriceps contribute to knee extension control and impact absorption, whereas the hamstrings can generate posterior tibial shear force, helping to constrain excessive anterior tibial translation and modulate ACL loading (DeMorat et al., 2004; Withrow et al., 2006; Shultz et al., 2015). These findings suggest that landing control depends on coordinated lower-limb muscle modules rather than isolated muscle activation (Jie et al., 2024). Therefore, examining the activation characteristics of the biceps femoris and semitendinosus/semimembranosus may help clarify how different hamstring components participate in coordinated knee joint control during landing.

Wavelet decomposition offers a promising method for in-depth analysis of hamstring activation characteristics. As a time-frequency analysis technique, wavelet decomposition can provide localized information in both the time and frequency domains, making it suitable for analyzing non-stationary EMG signals during dynamic movements such as landing (von Tscharner, 2000; Karlsson and Gerdle, 2001). Surface EMG power is mainly distributed within approximately 0–500 Hz, with dominant energy commonly observed around 50–150 Hz, whereas very low-frequency components are more susceptible to movement artifacts during dynamic tasks (De Luca et al., 2010). Therefore, compared with the 0–20 Hz band, the 60–200 Hz band is less likely to be dominated by landing-related motion artifacts, while still remaining close to the dominant frequency range of surface EMG. Physiologically, mid-to-high-frequency EMG components may be partly associated with the recruitment of faster motor units and changes in muscle fiber conduction velocity, which are relevant to rapid force production during landing (Kupa et al., 1995; Wakeling et al., 2002). However, because surface EMG reflects the summated activity of multiple motor units and is affected by electrode configuration, tissue filtering, movement artifacts, and other signal-processing factors, frequency-domain changes should be interpreted as indirect indicators of neuromuscular activation rather than direct evidence of specific muscle fiber-type recruitment (Farina et al., 2025). Functionally, the 60–200 Hz band may provide an indirect indicator of rapid hamstring neuromuscular activation during the landing load-acceptance phase, during which hamstring activation contributes to dynamic knee joint control and stability. Using this frequency band, wavelet decomposition may help reveal subtle changes in the activation characteristics of the biceps femoris and semitendinosus/semimembranosus that may not be captured by traditional time-domain EMG measures alone (Wakeling and Rozitis, 2004).

Therefore, the purpose of the present study was to investigate the acute effects of a hamstring conditioning contraction on neuromuscular activation characteristics and knee joint biomechanics during landing in male university athletes using a randomized controlled design. It was hypothesized that participants receiving the hamstring conditioning contraction would demonstrate greater increases in wavelet-derived EMG power within the 60–200 Hz frequency band of the hamstring muscles compared with the control condition. No *a priori* directional hypotheses were specified for secondary outcomes, which were analyzed in a supportive and exploratory manner.

## 2 Methods

### 2.1 Study design

This study was a two-arm, parallel-group randomized controlled trial with a 1:1 allocation ratio. The trial examined the acute effects of a hamstring conditioning contraction on neuromuscular activation characteristics and knee joint biomechanics during a standardized landing task. Participants completed baseline (pre-test) assessments followed by either a control condition or a hamstring MVIC conditioning protocol, and subsequently completed post-test assessments.

### 2.2 Participants

The minimum sample size was estimated using G*Power (version 3.0.10) for a two-group, two-time-point repeated-measures design, with an assumed effect size of f = 0.25, power = 0.80, and α = 0.05 (Bocheng et al., 2024). The calculation indicated that at least 17 participants per group were required to achieve adequate statistical power.

Male university athletes were recruited from a single university (Soochow University). The recruitment period lasted 1 week, and the experimental procedures were conducted from 10 February 2025 to 10 March 2025. Athletes were recruited from university sports teams including sprint, football, basketball, and high jump.

**FIGURE 1:**
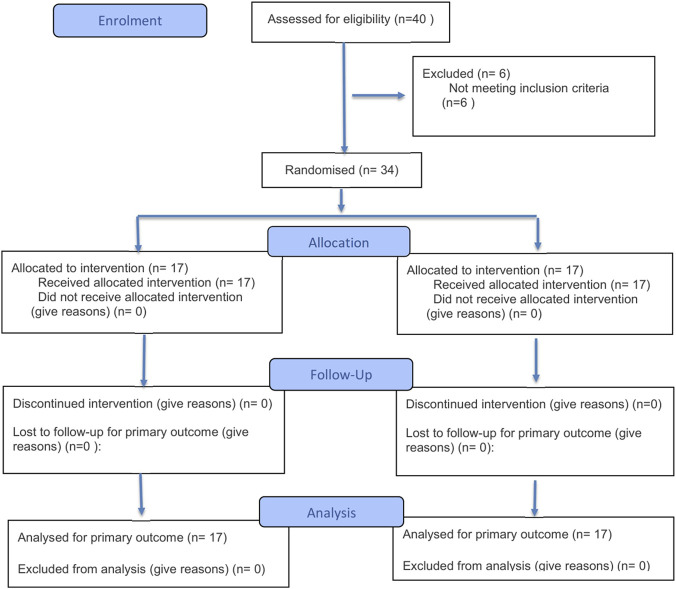
CONSORT flow diagram.

A total of 40 athletes were assessed for eligibility. Six athletes were excluded prior to randomization due to unexplained muscle pain (n = 1) or scheduling unavailability (n = 5). The remaining 34 eligible participants were randomized in a 1:1 ratio to a control group (n = 17) or a PAP group (n = 17). Male collegiate athletes were recruited to ensure a homogeneous training background and to minimize variability associated with sex-related neuromuscular and biomechanical differences during landing tasks. This population also represents individuals who regularly perform high-impact movements, making them suitable for examining acute neuromuscular responses to conditioning contractions. All randomized participants completed the study procedures and were included in the final analyses. The flow of participants through each stage of the study is presented in the CONSORT flow diagram (Figure 1).

The inclusion criteria for participants were as follows:

- Male athletes who are members of the university sports team and have participated in regular lower-limb strength training for the past 6 months;
- Right-leg dominance, determined using the kicking test, was required for inclusion. The dominant leg was defined as the leg that a participant naturally used to kick a ball with maximal force;
- No history of lower-limb neurological, muscular, or skeletal injuries in the past year;
- Mentally stable, without any psychiatric or psychological disorders;
- Voluntary participation in the study with a commitment to attend all interventions and evaluations on time.

Exclusion criteria included:

- Participants who were unwilling to continue in the study;
- Participants who sustained any injuries during the experimental period that would interfere with the intervention or assessments (e.g., ankle fractures, ACL tears, or meniscus tears).

Detailed characteristics of the participants are shown in Table 1.

**TABLE 1:** Participant characteristics.

Specific characteristic	Control group (n = 17)	PAP group (n = 17)
Age (years)	21.71 ± 3.21	21.89 ± 2.18
Height (m)	1.94 ± 0.07	1.90 ± 0.06
Weight (kg)	87.46 ± 5.39	88.14 ± 5.93
BMI(kg/m2)	23.24 ± 3.22	24.42 ± 4.27
Duration of lower-limb strength training (months)	13 ± 4.80	13 ± 3.70
Type of Sport	Sprint (n = 5); football (n = 5); basketball (n = 3); high jump (n = 4)	Sprint (n = 4); football (n = 5); basketball (n = 6); high jump (n = 2)

Baseline characteristics of the participants are presented in Table 1 Participant Characteristics. No statistically or clinically meaningful differences were observed between groups at baseline, indicating good comparability between the PAP and control groups prior to the intervention.

### 2.3 Experimental procedure

This study employed a randomized controlled design. Randomization was performed using a computer-generated random number sequence created in Microsoft Excel by an investigator who was not involved in participant recruitment, assessment, or data analysis. Participant enrollment was conducted by a separate investigator responsible for screening eligibility and obtaining informed consent. After baseline assessments were completed, group assignments were implemented by a third researcher who had access to the randomization list. The randomization list was accessible only to the researcher responsible for assignment and was not available to outcome assessors or data analysts. Given the laboratory-based design and the short intervention period, formal allocation concealment was not applied. Blinding was also not feasible because the intervention procedures were readily identifiable to both participants and investigators.

All participants were instructed to refrain from engaging in strenuous lower-limb activities for at least 48 h prior to testing and were required to maintain their habitual dietary routines throughout the experimental period. Each participant performed three landing trials at baseline and three additional trials following the intervention, with the landing height standardized at 50 cm. After the baseline landing trials, both groups underwent an 8-min interval before the post-test landing trials. During this interval, participants in the PAP group performed a 5-s hamstring Maximum Voluntary Isometric Contraction (MVIC) followed by the remaining rest period, whereas participants in the control group remained seated quietly for an equivalent duration without receiving any conditioning contraction. Therefore, the control condition was time-matched to the PAP condition, and the between-group comparison was designed to isolate the effect of the hamstring conditioning contraction rather than the effect of additional rest time.

During all landing tests, kinetic data were obtained using a three-dimensional force platform, kinematic parameters were recorded with a high-speed infrared camera system, and surface electromyography (sEMG) signals were collected to monitor the activation of the hamstrings and quadriceps. The study was conducted in accordance with the Declaration of Helsinki and was approved by the Ethics Review Board of Soochow University (approval number: SUDA20211227H03). Written informed consent was obtained from all participants prior to enrollment. This study was not registered in a clinical trial registry because it was not a clinical trial and involved only healthy athletes in a laboratory-based experimental setting.

#### 2.3.1 Landing test procedure

During the landing test, participants stood on a 50-cm-high step with both hands placed on the iliac crests. The 50-cm drop height was selected to provide a sufficiently challenging landing condition capable of eliciting measurable knee joint loading and hamstring neuromuscular responses, as comparable drop heights have been used in previous landing-biomechanics studies examining ACL-related risk factors and lower-limb muscle activation (Ha and Park, 2018; Sahabuddin et al., 2021).

**FIGURE 2:**
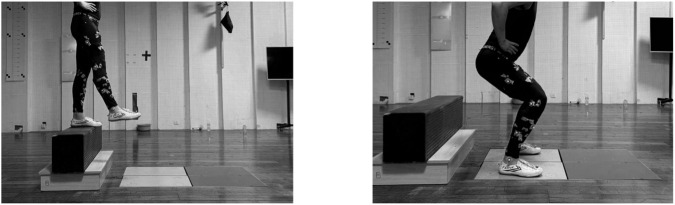
Landing test motion.

Because all participants were right-leg dominant, they extended the dominant right leg forward off the step while the left leg pushed off to initiate landing, ensuring that both feet contacted the centers of two separate force platforms simultaneously. After a brief stabilization period, participants were required to maintain an upright posture to complete the trial. This procedure was performed three times during the baseline session and repeated three times during the post-test session. For the control group, the post-test served as a repeated assessment without intervention, whereas the PAP group underwent the landing test again after completing the MVIC protocol. All participants successfully completed the standardized landing task, and no participant was excluded because of failure to perform the experimental movement. The landing test motion is shown in Figure 2.

#### 2.3.2 MVIC intervention protocol

For participants allocated to the PAP group, the intervention consisted of Maximum Voluntary Isometric Contraction (MVIC) of the hamstrings performed in a prone position with both knees naturally flexed. Two experimenters provided manual resistance against knee flexion to ensure maximal contraction while maintaining a constant joint angle. Participants in the PAP group performed a 5-s MVIC and then rested for 8 min before the post-test. Participants in the control group did not receive any conditioning contraction but remained seated quietly for an equivalent interval before the post-test. Thus, both groups had the same recovery/waiting period before the post-test landing trials.

#### 2.3.3 Landing biomechanics testing

The landing biomechanics were recorded using the Vicon infrared high-speed motion capture system (Vicon, Oxford, UK) and Kistler force platforms (Kistler, Winterthur, Switzerland). The cameras had a sampling frequency of 200 Hz, and the force platforms had a sampling frequency of 1000 Hz.

Participants wore tight-fitting clothing and were familiarized with the testing equipment and procedures before the test. After a standard warm-up, markers (Mark balls) were applied to the participants’ lower limbs to create a model of the lower body. The marker positions were as follows: bilateral anterior superior iliac spines (ASIS), bilateral posterior superior iliac spines (PSIS), bilateral lateral femoral condyles, bilateral medial femoral condyles, bilateral external and internal malleoli, bilateral calcaneus, bilateral second metatarsal heads, left thigh and calf at 1/3 and 2/3 of the length, right thigh and calf at 1/3 and 2/3 of the length, and the lateral midpoint of the left and right thighs. To minimize experimental errors, all marker placements were performed by a single experimenter.

The Vicon system was activated, and participants stood on the force platforms with arms extended, maintaining a static position while collecting the static model data. After the static data collection, landing biomechanics were recorded during the drop-landing task. The landing absorption phase was subsequently extracted for analysis. Each participant performed three baseline landing tests and three post-intervention landing tests. The second trial was used as the final evaluation. If the second trial was unavailable, the first or third trial results were used for analysis.

Outcome measures included: maximum knee joint angles (°) in the X, Y, and Z-axes; peak moment (N·m/kg) in the X, Y, and Z-axes during knee flexion up to 40° in the X-axis; moment (N·m/kg) at the maximum knee flexion angle in the X, Y, and Z-axes; and peak moment (N·m/kg) in the X, Y, and Z-axes.

#### 2.3.4 Surface electromyography (sEMG) testing

Throughout the experiment, the YW-Wireless wireless surface electromyography system (made in China, sampling frequency 1000 Hz) was used to monitor the electromyographic signals from the vastus lateralis, rectus femoris, vastus medialis, and hamstrings (including biceps femoris and semitendinosus/semimembranosus). This system was synchronized with the Vicon 3D motion capture system via hardware to ensure temporal alignment of the EMG, kinematic, and kinetic data.

One day before the experiment, maximum voluntary isometric contraction (MVIC) tests were performed on the vastus lateralis, rectus femoris, vastus medialis, biceps femoris, and semitendinosus/semimembranosus to normalize the surface EMG data. The MVIC test required participants to perform maximal isometric contractions of the target muscles. Two experimenters provided resistance to the participant’s maximal knee flexion while instructing them to contract the muscles at maximal force for at least 10 s. Surface EMG signals were collected from the right lower limb, which corresponded to the dominant leg in all participants. Prior to electrode placement, the skin was lightly abraded with sandpaper and cleaned with alcohol to ensure optimal signal quality. The specific electrode placements and MVC testing methods are shown in Table 2.

**TABLE 2:** Surface electromyography sensor placement and MVIC testing methods.

Muscle name	Sensor placement	MVIC testing method
Vastus lateralis	On the lateral side of the thigh, at the highest point of muscle bulge above the anterior iliac spine	The participant sits at the edge of a chair with the knee flexed at 70°–80°. Resistance is applied above the ankle, and the knee performs an isometric extension against resistance
Rectus femoris	On the front of the thigh, at the highest point of the muscle bulge along the line connecting the anterior superior iliac spine (ASIS) to the base of the patella	Same as for vastus lateralis
Vastus medialis	On the medial side of the thigh, at the highest point of the muscle bulge near the anterior iliac spine	Same as for vastus lateralis
Biceps femoris	On the posterior side of the thigh, at the highest point of the muscle bulge along the line connecting the ischial tuberosity to the lateral condyle of the tibia	The participant lies prone with the knee flexed at 45°. Resistance is applied to the distal calf near the calcaneus, and the knee performs an isometric flexion against resistance
Semitendinosus	On the posterior side of the thigh, at the highest point of the muscle bulge along the line connecting the ischial tuberosity to the medial condyle of the tibia	Same as for biceps femoris

All muscle electrode placements were standardized according to the SENIAM (Surface Electromyography for the Non-Invasive Assessment of Muscles) guidelines (Hermens et al., 2000).

#### 2.3.5 Data processing and analysis

The motion capture data from the Vicon system, including the trajectories of reflective markers, were processed using Nexus software (version 2.7.1). The kinematic and dynamic data were then imported into Visual3D (C-Motion, Germantown, MD, United States of America, version 5.02.3). A static skeletal model with nine segments was created based on the static file. In Visual3D, the kinematic and dynamic data were smoothed using a second-order bidirectional Butterworth low-pass filter, with cutoff frequencies of 6 Hz and 20 Hz. The knee joint’s 3D angular changes were computed using Cardan angles, and the 3D knee joint torque was calculated using inverse dynamics algorithms.

The following definitions were used for the knee joint movement: the X-axis was defined as knee extension (+)/flexion (−), the Y-axis as knee adduction (−)/abduction (+), and the Z-axis as knee external rotation (−)/internal rotation (+).

The landing absorption phase was defined based on the vertical ground reaction force (vGRF) and knee flexion angle. Initial contact was defined as the first frame in which the vGRF exceeded 10 N. The time of maximum knee flexion was defined as the frame at which the knee flexion angle reached its maximum value along the X-axis. The landing absorption phase was defined as the period from initial contact to maximum knee flexion. All biomechanical and EMG outcomes related to landing were extracted from this phase unless otherwise specified.

Surface electromyography (sEMG) signals were collected using the YW-Wireless wireless EMG acquisition system. During signal acquisition, synchronization with the Vicon motion capture system was ensured through hardware to maintain consistency between the motion and EMG data. To eliminate noise and retain relevant EMG information, all EMG signals underwent preprocessing using a bandpass filter. A second-order Butterworth bandpass filter was applied, with a frequency range of 10–480 Hz, and a 50 Hz notch filter was used to eliminate power line interference. The filtering process was implemented in Matlab 2021a, and the signals were smoothed using the Butterworth filter. To eliminate individual differences between subjects, the EMG signals were normalized. The normalization method involved calculating the ratio of the iEMG during the landing absorption phase to the iEMG of the muscle’s maximum voluntary isometric contraction (MVIC).

The knee flexor–extensor co-activation ratio was calculated from normalized iEMG values during the landing absorption phase, rather than from peak EMG or an overlap integral. The formula was:

$$Co-activation\;ratio=\frac{{iEMG}_{BF}+{iEMG}_{ST/SM}}{{iEMG}_{VL}+{iEMG}_{RF}+iEMG_{VM}}$$

BF, ST/SM, VL, RF, and VM represent the biceps femoris, semitendinosus/semimembranosus, vastus lateralis, rectus femoris, and vastus medialis, respectively.

For the hamstrings (including the biceps femoris and semitendinosus/semimembranosus), wavelet decomposition was performed using the Amor wavelet. For descriptive time-frequency analysis, the wavelet-derived surface EMG spectrum was divided into four predefined frequency bands: 0–20 Hz, 20–60 Hz, 60–200 Hz, and 200–480 Hz. These bands were used to characterize the distribution of surface EMG power across low-, low-to-middle-, middle-to-high-, and high-frequency ranges, respectively. Because surface EMG frequency content can be influenced by multiple physiological and non-physiological factors, including motor unit activity, muscle fiber conduction velocity, tissue filtering, electrode placement, and movement artifacts, these frequency bands were interpreted as descriptive surface EMG features rather than direct indicators of specific neural or muscle-fiber mechanisms.

### 2.4 Outcomes

The pre-specified primary outcomes were wavelet-derived EMG power values in the 60–200 Hz frequency band for the semitendinosus/semimembranosus and biceps femoris during the landing absorption phase.

Secondary outcomes included wavelet-derived EMG power in other frequency bands (0–20, 20–60, and 200–480 Hz), knee joint kinematics and kinetics during landing, integrated EMG (iEMG), and the hamstring-to-quadriceps co-activation ratio.

### 2.5 Statistical analysis

All statistical analyses were conducted using SPSS 26.0 (IBM Corp., Armonk, NY, United States of America). The distribution of each continuous variable was assessed using the Shapiro–Wilk test, together with visual inspection of histograms and Q–Q plots. Normally distributed variables are presented as mean ± standard deviation, whereas non-normally distributed variables are presented as median and interquartile range [median (IQR)].

For the wavelet-derived EMG power variables, several frequency-band outcomes showed clear deviations from normality. Therefore, nonparametric tests were used for the wavelet-band analyses. Within-group pre–post comparisons were performed using the Wilcoxon signed-rank test, and between-group comparisons at pre- and post-intervention were performed using the Mann–Whitney U test. Effect sizes for nonparametric comparisons were reported as rank-biserial correlations. Hodges–Lehmann estimates and their 95% confidence intervals were calculated to quantify the magnitude of the median difference.

For normally distributed biomechanical and time-domain EMG variables, a two-way repeated-measures analysis of variance (ANOVA) was used, with group (control vs. PAP) as the between-subject factor and time (pre-test vs. post-test) as the within-subject factor. When a significant group × time interaction was observed, simple-effect analyses were conducted to examine within-group pre–post changes and between-group differences at each time point. Partial eta squared (ηp^2^) was reported as the effect size for ANOVA results.

To address the risk of inflated type I error due to multiple statistical comparisons, all p values generated from the primary, secondary, and exploratory analyses were pooled and adjusted together using the Benjamini–Hochberg false discovery rate (FDR) procedure. Both unadjusted and FDR-adjusted p values were examined, but statistical interpretation was based primarily on the FDR-adjusted p values.

To distinguish confirmatory from exploratory analyses, an *a priori* analysis hierarchy was established. The primary outcomes were wavelet-derived EMG power values within the 60–200 Hz frequency band for the biceps femoris and semitendinosus/semimembranosus during the landing absorption phase. Confirmatory analyses for these primary outcomes focused on within-group pre–post changes and post-intervention between-group differences. All other EMG frequency bands, biomechanical variables, integrated EMG measures, and co-activation ratios were classified as secondary or exploratory outcomes and were interpreted cautiously as hypothesis-generating findings. The significance level was set at α = 0.05.

## 3 Results

### 3.1 Adverse events

All primary and secondary outcomes were analyzed using data from all 34 participants (17 per group). No missing data were observed for any outcome measures, and no participants were excluded from the analyses.

No adverse events or injuries were reported during the intervention or testing procedures in either group.

### 3.2 Primary outcomes

The pre-specified primary outcomes were wavelet-derived EMG power values in the 60–200 Hz frequency band for the semitendinosus/semimembranosus and biceps femoris during the landing absorption phase. Because the wavelet-band power values did not satisfy the assumption of normality in several conditions, nonparametric analyses were performed. Confirmatory analyses focused on within-group pre–post changes and post-intervention between-group comparisons. Data are presented as median and interquartile range.

#### 3.2.1 Semitendinosus/semimembranosus in the 60–200 Hz frequency band

**FIGURE 3:**
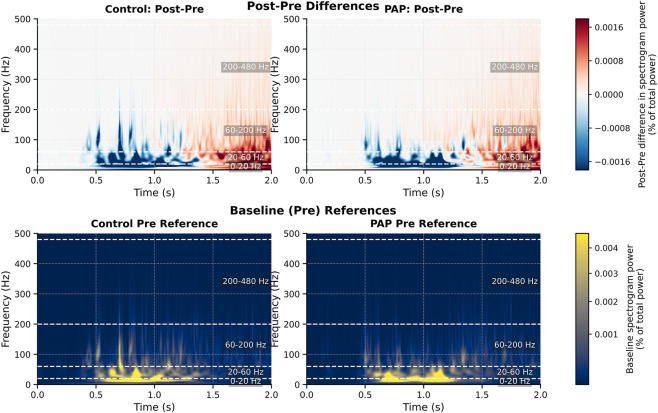
Time–frequency representations of EMG power of semitendinosus/semimembranosus muscle.

For the semitendinosus/semimembranosus, the PAP group showed a significant increase in 60–200 Hz wavelet power from pre-to post-intervention, whereas no significant change was observed in the control group. Specifically, the PAP group increased from 2261.641 [836.862 to 2749.885] to 4226.650 [2855.625 to 9508.892] μV^2^ (Wilcoxon signed-rank test, FDR-adjusted p = 0.002, rank-biserial effect = 0.682, HL estimate = 1192.514, 95% CI: 927.318–2267.518). The control group showed no significant pre–post change (FDR-adjusted p = 0.633). Post-intervention between-group comparison further indicated significantly greater 60–200 Hz wavelet power in the PAP group than in the control group (Mann–Whitney U test, FDR-adjusted p = 0.014, rank-biserial effect = 0.265, HL estimate = 3347.459, 95% CI: 2100.466–6465.455). Specific results are shown in Table 3 and Figure 3.

**TABLE 3:** Wavelet-derived EMG power during landing.

Muscle	Band	Control pre (μV^2^)	Control post (μV^2^)	PAP pre (μV^2^)	PAP post (μV^2^)
ST/SM	0–20 Hz	23870.930 [13697.839–48657.587]	31944.316 [2434.211–46339.666]	19897.130 [12157.660–37953.903]	12301.576 [1891.165–46454.003]
	20–60 Hz	33416.959 [12386.095–52289.884]	32986.408 [13775.569–82044.820]	22637.927 [902.912–34208.435]	44136.420 [20167.166–74161.821]
	60–200 Hz#	1895.716 [1463.310–5492.004]	2581.472 [1227.664–8611.527]	2261.641 [836.862–2749.885]	4226.650 [2855.625–9508.892]
	200–480 Hz	182.085 [36.921–239.063]	166.099 [36.595–487.863]	192.338 [26.577–363.973]	482.666 [148.870–697.938]
BF	0–20 Hz	35153.841 [13387.807–41089.738]	27750.879 [12815.573–51476.869]	25086.512 [5973.817–33883.767]	21359.395 [3459.858–32453.702]
	20–60 Hz	12507.151 [7675.718–23475.033]	14935.948 [8092.172–17466.178]	12396.673 [9870.590–16122.677]	11436.868 [9904.197–27923.046]
	60–200 Hz#	1929.519 [1335.558–2908.166]	2043.650 [1450.036–3220.686]	2120.429 [1154.748–2765.458]	4289.574 [1663.306–5898.108]
	200–480 Hz	85.548 [67.586–154.195]	108.113 [73.206–203.996]	126.094 [63.160–149.155]	133.914 [121.284–432.304]

Values are presented as median [interquartile range]. ST/SM, semitendinosus/semimembranosus; BF, biceps femoris; PAP, post-activation potentiation. #Pre-specified primary outcome.

#### 3.2.2 Biceps femoris in the 60–200 Hz frequency band

**FIGURE 4:**
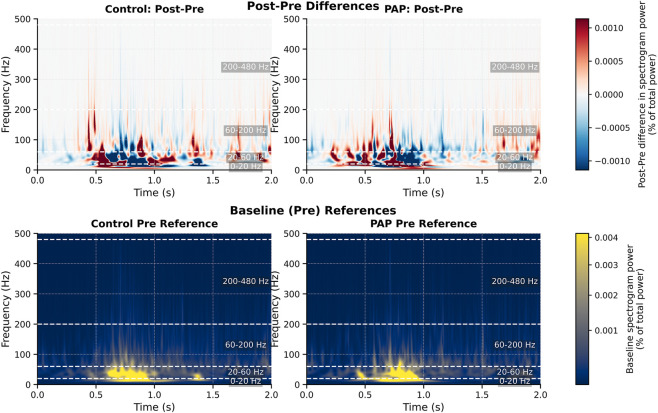
Time–frequency representations of EMG power of biceps femoris muscle.

For the biceps femoris, the PAP group also demonstrated a significant increase in 60–200 Hz wavelet power from pre-to post-intervention, increasing from 2120.429 [1154.748 to 2765.458] to 4289.574 [1663.306 to 5898.108] μV^2^ (Wilcoxon signed-rank test, FDR-adjusted p = 0.002, rank-biserial effect = 0.746, HL estimate = 958.932, 95% CI: 436.374–1615.918). No significant pre–post change was observed in the control group (FDR-adjusted p = 0.602). At post-intervention, the PAP group showed significantly higher 60–200 Hz wavelet power than the control group (Mann–Whitney U test, FDR-adjusted p = 0.045, rank-biserial effect = 0.336, HL estimate = 845.924, 95% CI: 605.924–1131.846). The detailed results are shown in Table 4 and Figure 4.

**TABLE 4:** Nonparametric comparisons of wavelet-derived EMG.

Outcome	Comparison	Test	FDR p value	Rank-biserial effect	HL estimate	95% CI
Primary outcomes						
ST/SM, 60–200 Hz	Control post vs. pre	Wilcoxon signed-rank	0.633	0.024	313.381	−1097.470 to 398.333
	PAP post vs. pre	Wilcoxon signed-rank	0.002	0.682	1192.514	927.318 to 2267.518
	PAP vs. control at pre	Mann–Whitney U	0.602	0.114	73.977	−1280.378 to 669.264
	PAP vs. control at post	Mann–Whitney U	0.014	0.265	3347.459	2100.466 to 6465.455
BF, 60–200 Hz	Control post vs. pre	Wilcoxon signed-rank	0.602	0.092	257.890	−34.231 to 701.031
	PAP post vs. pre	Wilcoxon signed-rank	0.002	0.746	958.932	436.374 to 1615.918
	PAP vs. control at pre	Mann–Whitney U	0.965	0.008	0.000	−569.488 to 696.192
	PAP vs. control at post	Mann–Whitney U	0.045	0.336	845.924	605.924 to 1131.846
Secondary/exploratory outcomes						
ST/SM, 0–20 Hz	Control post vs. pre	Wilcoxon signed-rank	0.680	0.022	3369.865	−823.082 to 9558.237
	PAP post vs. pre	Wilcoxon signed-rank	0.328	0.106	944.564	−40.652 to 11126.198
	PAP vs. control at pre	Mann–Whitney U	0.608	−0.111	−353.503	−6701.663 to 3.270
	PAP vs. control at post	Mann–Whitney U	0.135	−0.210	−7163.046	−22259.148 to 1477.849
ST/SM, 20–60 Hz	Control post vs. pre	Wilcoxon signed-rank	0.665	0.104	75.886	−4185.258 to 984.565
	PAP post vs. pre	Wilcoxon signed-rank	0.555	0.021	8158.613	−6954.212 to 9746.209
	PAP vs. control at pre	Mann–Whitney U	0.470	−0.147	−2858.673	−8514.047 to 3504.698
	PAP vs. control at post	Mann–Whitney U	0.954	−0.012	−10007.185	−280256.159 to 65914.357
ST/SM, 200–480 Hz	Control post vs. pre	Wilcoxon signed-rank	0.908	0.076	137.960	−58.763 to 255.836
	PAP post vs. pre	Wilcoxon signed-rank	0.002	0.587	108.983	89.751 to 152.967
	PAP vs. control at pre	Mann–Whitney U	0.634	0.103	21.189	−67.038 to 24.646
	PAP vs. control at post	Mann–Whitney U	0.042	0.312	103.039	5.197 to 246.071
BF, 0–20 Hz	Control post vs. pre	Wilcoxon signed-rank	0.815	0.074	382.190	−2935.555 to 2444.539
	PAP post vs. pre	Wilcoxon signed-rank	0.026	−0.556	−13350.581	−23341.855 to −1475.151
	PAP vs. control at pre	Mann–Whitney U	0.862	−0.041	−67.329	−2615.416 to 3043.674
	PAP vs. control at post	Mann–Whitney U	0.014	−0.536	−1163.754	−1976.941 to −933.626
BF, 20–60 Hz	Control post vs. pre	Wilcoxon signed-rank	0.744	−0.101	−565.258	−7918.338 to 1638.572
	PAP post vs. pre	Wilcoxon signed-rank	0.721	0.111	1040.517	−3115.598 to 7930.970
	PAP vs. control at pre	Mann–Whitney U	0.810	−0.058	−216.493	−6252.087 to 3369.441
	PAP vs. control at post	Mann–Whitney U	0.350	0.185	2029.507	−3121.309 to 7627.414
BF, 200–480 Hz	Control post vs. pre	Wilcoxon signed-rank	0.388	0.081	15.603	−0.621 to 64.857
	PAP post vs. pre	Wilcoxon signed-rank	0.049	0.276	89.171	6.920 to 165.899
	PAP vs. control at pre	Mann–Whitney U	0.910	0.025	0.000	−33.686 to 55.802
	PAP vs. control at post	Mann–Whitney U	0.032	0.360	33.310	1.276 to 121.833

Within-group pre–post comparisons were performed using the Wilcoxon signed-rank test, and between-group comparisons at each time point were performed using the Mann–Whitney U test. Effect sizes are reported as rank-biserial correlations. HL, Hodges–Lehmann estimate; CI, confidence interval; PAP, post-activation potentiation; ST/SM, semitendinosus/semimembranosus; BF, biceps femoris. The 60–200 Hz frequency band was defined *a priori* as the primary outcome; all other frequency bands were considered secondary or exploratory outcomes.

### 3.3 Secondary and exploratory outcomes

Secondary analyses examined wavelet-derived EMG power in the 0–20 Hz, 20–60 Hz, and 200–480 Hz frequency bands. These outcomes were interpreted as exploratory and supportive of the primary findings.

#### 3.3.1 Other EMG bands

##### 3.3.1.1 Semitendinosus/semimembranosus

For the semitendinosus/semimembranosus, no significant pre–post changes were observed in the control group across the secondary frequency bands. In the PAP group, no significant changes were observed in the 0–20 Hz band or the 20–60 Hz band. However, the PAP group showed a significant increase in the 200–480 Hz band from pre-to post-intervention, increasing from 192.338 [26.577 to 363.973] to 482.666 [148.870 to 697.938] μV^2^ (Wilcoxon signed-rank test, FDR-adjusted p = 0.002, rank-biserial effect = 0.587, HL estimate = 108.983, 95% CI: 89.751–152.967). Post-intervention between-group comparison also showed significantly higher 200–480 Hz wavelet power in the PAP group than in the control group (Mann–Whitney U test, FDR-adjusted p = 0.042, rank-biserial effect = 0.312, HL estimate = 103.039, 95% CI: 5.197–246.071).

Specific results are shown in Table 3 and Figure 3.

##### 3.3.1.2 Biceps femoris

For the biceps femoris, no significant pre–post changes were observed in the control group across any secondary frequency band. In the PAP group, significant exploratory changes were observed in the 0–20 Hz and 200–480 Hz bands, whereas no significant change was observed in the 20–60 Hz band.

In the 0–20 Hz band, the PAP group showed a significant decrease from pre-to post-intervention, with wavelet power changing from 25086.512 [5973.817 to 33883.767] to 21359.395 [3459.858 to 32453.702] μV^2^ (Wilcoxon signed-rank test, FDR-adjusted p = 0.026, rank-biserial effect = −0.556, HL estimate = −13350.581, 95% CI: −23341.855 to −1475.151). Post-intervention between-group comparison also indicated significantly lower 0–20 Hz wavelet power in the PAP group than in the control group (Mann–Whitney U test, FDR-adjusted p = 0.014, rank-biserial effect = −0.536, HL estimate = −1163.754, 95% CI: −1976.941 to −933.626).

In the 200–480 Hz band, the PAP group increased from 126.094 [63.160 to 149.155] to 133.914 [121.284 to 432.304] μV^2^ (Wilcoxon signed-rank test, FDR-adjusted p = 0.049, rank-biserial effect = 0.276, HL estimate = 89.171, 95% CI: 6.920–165.899). The post-intervention between-group comparison was also significant, with higher 200–480 Hz wavelet power in the PAP group than in the control group (Mann–Whitney U test, FDR-adjusted p = 0.032, rank-biserial effect = 0.360, HL estimate = 33.310, 95% CI: 1.276–121.833). No significant change was observed in the 20–60 Hz band.

The detailed results are shown in Table 4 and Figure 4.

#### 3.3.2 Landing biomechanics test results

##### 3.3.2.1 Maximum knee motion angles

Repeated-measures ANOVA revealed significant main effects of group (F = 185.513, FDR-adjusted p = 0.002, ηp^2^ = 0.746), time (F = 23.750, FDR-adjusted p = 0.002, ηp^2^ = 0.430), and their interaction (F = 46.325, FDR-adjusted p = 0.002, ηp^2^ = 0.595) for the X-axis knee angle. Simple effects analysis showed that post-intervention, the PAP group exhibited significantly greater maximal X-axis knee angles than both the control group (p < 0.001, 95% CI [6.374, 10.699]) and its own pre-intervention values (p = 0.002, 95% CI [4.338, 10.507]).

For the Y-axis knee angle, significant main effects of group (F = 25.747, FDR-adjusted p = 0.002, ηp^2^ = 0.290), time (F = 5.572, FDR-adjusted p = 0.014, ηp^2^ = 0.150), and interaction (F = 4.262, FDR-adjusted p = 0.037, ηp^2^ = 0.119) were observed. Post-intervention, the PAP group showed significantly smaller maximal Y-axis angles than the control group (p < 0.001, 95% CI [−9.627, −3.554]) and pre-intervention (p = 0.001, 95% CI [−8.341, −1.814]).

For the Z-axis knee angle, ANOVA showed significant main effects of group (F = 14.465, FDR-adjusted p = 0.002, ηp^2^ = 0.187), time (F = 20.836, FDR-adjusted p = 0.002, ηp^2^ = 0.398), and interaction (F = 17.976, FDR-adjusted p = 0.002, ηp^2^ = 0.363). Post-intervention, the PAP group had significantly smaller maximal Z-axis knee angles compared with the control group (p < 0.001,95%CI [-10.227, −5.737]) and the respective pre-intervention values (p < 0.001,95%CI [-6.155, −2.693]).

**FIGURE 5:**
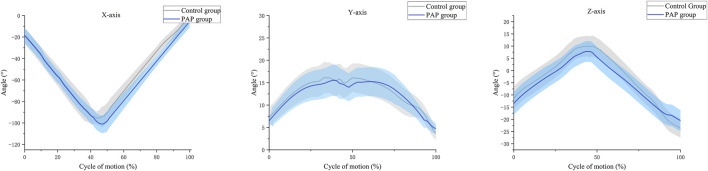
Post-intervention knee angle variations during the landing absorption phase.

Detailed results are presented in Table 5, and the angular changes during the landing absorption phase are shown in Figure 5.

**TABLE 5:** Knee motion angles.

Angle of motion	Time	Control group (°)	PAP group (°)	ANOVA		
				F	FDR P	pη^2^
X-axis	Pre-intervention	−89.85 ± 3.79	−89.88 ± 2.24	​	​	​
	Post-intervention	−90.65 ± 4.53	−98.60 ± 2.67	​	​	​
	Group main effect	​	​	185.513	0.002	0.746
	Time main effect	​	​	23.750	0.002	0.430
	Interactive effect	​	​	46.325	0.002	0.595
Y-axis	Pre-intervention	21.39 ± 4.94	22.13 ± 6.59	​	​	​
	Post-intervention	21.73 ± 5.72	16.48 ± 2.89	​	​	​
	Group main effect	​	​	25.764	0.002	0.290
	Time main effect	​	​	5.572	0.014	0.150
	Interactive effect	​	​	4.262	0.037	0.119
Z-axis	Pre-intervention	19.01 ± 2.38	18.58 ± 3.10	​	​	​
	Post-intervention	20.96 ± 2.95	15.44 ± 4.01	​	​	​
	Group main effect	​	​	14.465	0.002	0.187
	Time main effect	​	​	20.836	0.002	0.398
	Interactive effect	​	​	17.976	0.002	0.363

##### 3.3.2.2 Maximum moment in 40° of knee flexion

The Shapiro-Wilk test confirmed that the maximum moment values along the X, Y, and Z-axes within 40° of knee flexion followed a normal distribution.

For the X-axis joint moment, repeated-measures ANOVA showed a nonsignificant main effect of group (F = 1.796, FDR-adjusted p = 0.267, ηp^2^ = 0.028), a significant main effect of time (F = 12.701, FDR-adjusted p = 0.002, ηp^2^ = 0.287), and a significant interaction effect (F = 8.987, FDR-adjusted p = 0.002, ηp^2^ = 0.222). Simple effects analysis revealed that, post-intervention, the PAP group exhibited significantly lower first-peak joint moments than both the control group (p < 0.001, 95% CI [−0.743, −0.249]) and its own pre-intervention values (p < 0.001, 95% CI [−5.322, −1.861]).

For the Y-axis joint moment, ANOVA indicated a significant main effect of group (F = 7.659, FDR-adjusted p = 0.016, ηp^2^ = 0.108), a nonsignificant main effect of time (F = 0.402, FDR-adjusted p = 0.761, ηp^2^ = 0.013), and a significant interaction effect (F = 5.311, FDR-adjusted p = 0.016, ηp^2^ = 0.144). Post-intervention, the PAP group showed significantly lower first-peak joint moments than the control group (p = 0.005, 95% CI [−0.247, −0.110]) and pre-intervention (p = 0.045, 95% CI [−0.311, −0.021]).

For the Z-axis joint moment, significant main effects of group (F = 14.465, FDR-adjusted p = 0.002, ηp^2^ = 0.187), main effects of time (F = 20.836, FDR-adjusted p = 0.002, ηp^2^ = 0.398) and interaction (F = 17.976, FDR-adjusted p = 0.002, ηp^2^ = 0.363) were observed. Simple effects analysis showed that, post-intervention, the PAP group had significantly lower first-peak joint moments than both the control group (p < 0.001, 95% CI [−0.376, −0.165]) and pre-intervention (p < 0.001, 95% CI [−0.343, −0.158]).

The detailed results are presented in Table 6.

**TABLE 6:** Maximum joint moment within 40° of knee flexion.

Joint moment	Time	Control group(N·m/kg)	PAP group(N·m/kg)	ANOVA		
				F	FDR P	pη^2^
X-axis	Pre-intervention	1.22 ± 0.33	1.13 ± 0.45	​	​	​
	Post-intervention	1.27 ± 0.48	0.96 ± 0.24	​	​	​
	Group main effect	​	​	1.796	0.267	0.028
	Time main effect	​	​	12.701	0.002	0.287
	Interactive effect	​	​	8.987	0.002	0.222
Y-axis	Pre-intervention	0.69 ± 0.35	0.72 ± 0.27	​	​	​
	Post-intervention	0.72 ± 0.31	0.56 ± 0.18	​	​	​
	Group main effect	​	​	7.659	0.016	0.108
	Time main effect	​	​	0.402	0.761	0.013
	Interactive effect	​	​	5.311	0.016	0.144
Z-axis	Pre-intervention	0.44 ± 0.18	0.51 ± 0.10	​	​	​
	Post-intervention	0.46 ± 0.25	0.34 ± 0.29	​	​	​
	Group main effect	​	​	8.155	0.002	0.129
	Time main effect	​	​	12.817	0.002	0.353
	Interactive effect	​	​	10.164	0.002	0.312

##### 3.3.2.3 Maximum knee moment

The Shapiro-Wilk test confirmed that the maximum knee joint moments along the X, Y, and Z-axes followed a normal distribution.

For the X-axis peak knee joint moment, repeated-measures ANOVA revealed significant main effects of group (F = 49.890, FDR-adjusted p = 0.002, ηp^2^ = 0.442) and time (F = 4.829, FDR-adjusted p = 0.024, ηp^2^ = 0.133), as well as a significant group × time interaction (F = 4.037, FDR-adjusted p = 0.044, ηp^2^ = 0.114). Subsequent simple effects analyses indicated that, at post-intervention, the PAP group exhibited significantly lower peak X-axis knee joint moments compared with the control group (p = 0.048, 95% CI [−0.408, −0.015]). In addition, within-group comparisons showed a significant reduction in peak X-axis moment from pre-to post-intervention in the PAP group (p = 0.003, 95% CI [−0.469, −0.100]), whereas no significant change was observed in the control group.

For the Y-axis peak knee joint moment, ANOVA indicated a significant group main effect (F = 20.039, FDR-adjusted p = 0.002, ηp^2^ = 0.241) and interaction effect (F = 5.447, FDR-adjusted p = 0.014, ηp^2^ = 0.148), whereas the time main effect (F = 2.393, FDR-adjusted p = 0.151, ηp^2^ = 0.071) were nonsignificant. Simple effects analysis showed that, post-intervention, the PAP group exhibited significantly lower peak Y-axis moments than the control group (p = 0.043, 95% CI [−0.323, −0.010]) and pre-intervention (p = 0.049, 95% CI [−0.306, −0.006]).

For the Z-axis peak knee joint moment, significant main effects of group (F = 21.574, FDR-adjusted p = 0.002, ηp^2^ = 0.255), time (F = 5.238, FDR-adjusted p = 0.018, ηp^2^ = 0.143), and interaction (F = 9.435, FDR-adjusted p = 0.002, ηp^2^ = 0.230) were found. Simple effects analysis indicated that, post-intervention, the PAP group exhibited significantly lower peak Z-axis moments than both the control group (p = 0.002, 95% CI [−0.360, −0.068]) and pre-intervention (p = 0.002, 95% CI [−0.320, −0.079]).

**FIGURE 6:**
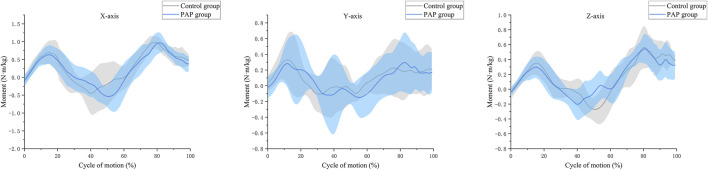
Post-intervention variations in knee moments during the landing absorption phase.

Detailed results are presented in Table 7, and the moment changes during the landing absorption phase are shown in Figure 6.

**TABLE 7:** Maximum knee moment.

Joint moment	Time	Control group(N·m/kg)	PAP group(N·m/kg)	ANOVA		
				F	FDR P	pη^2^
X-axis	Pre-intervention	1.47 ± 0.32	1.39 ± 0.41	​	​	​
	Post-intervention	1.40 ± 0.38	1.15 ± 0.34	​	​	​
	Group main effect	​	​	49.890	0.002	0.442
	Time main effect	​	​	4.829	0.024	0.133
	Interactive effect	​	​	4.037	0.044	0.114
Y-axis	Pre-intervention	0.87 ± 0.28	0.91 ± 0.33	​	​	​
	Post-intervention	0.88 ± 0.28	0.65 ± 0.19	​	​	​
	Group main effect	​	​	20.439	0.002	0.241
	Time main effect	​	​	2.391	0.151	0.071
	Interactive effect	​	​	5.477	0.014	0.148
Z-axis	Pre-intervention	0.86 ± 0.30	0.90 ± 0.13	​	​	​
	Post-intervention	0.88 ± 0.23	0.70 ± 0.21	​	​	​
	Group main effect	​	​	21.574	0.002	0.255
	Time main effect	​	​	5.238	0.018	0.143
	Interactive effect	​	​	9.435	0.002	0.230

#### 3.3.3 iEMG

The Shapiro-Wilk test indicated that the iEMG data for each muscle during the landing absorption phase followed a normal distribution.

The repeated measures ANOVA of the vastus lateralis iEMG revealed that the main effects of time and group, as well as their interaction, were not significant.

The repeated measures ANOVA of the rectus femoris iEMG revealed that the main effects of time and group, as well as their interaction, were not significant.

The repeated measures ANOVA of the vastus medialis iEMG revealed that the main effects of time and group, as well as their interaction, were not significant.

The repeated measures ANOVA of the semitendinosus/semitendinosus iEMG revealed that the main effects of time and group, as well as their interaction, were not significant.

The repeated measures ANOVA of the biceps femoris iEMG showed a significant main effect of group (F = 5.153, FDR-adjusted p = 0.035, pη^2^ = 0.213) and a significant interaction effect (F = 3.130, FDR-adjusted p = 0.036, pη^2^ = 0.163). Simple effect analysis indicated that, after the intervention, the iEMG of the biceps femoris in the PAP group was significantly higher than both pre-intervention (p = 0.001, 95% CI [0.027, 0.110]) and the control group (p = 0.001, 95% CI [0.025, 0.114]).

**FIGURE 7:**
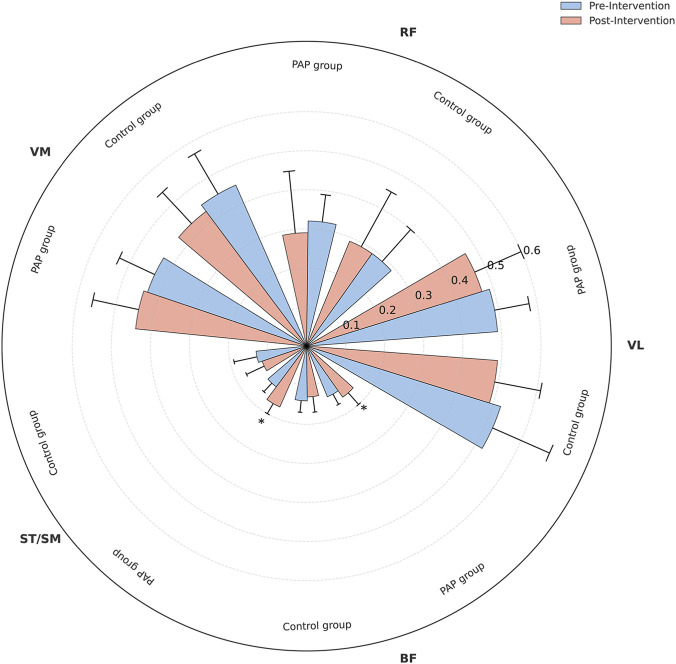
Pre- and Post-Intervention Comparison of iEMG of Lower Limb Muscles.

The detailed results are shown in Table 8 and Figure 7.

**TABLE 8:** Pre- and Post-Intervention iEMG Results of Lower Limb Muscles.

Muscle	Time	Control group	PAP group	ANOVA		
				F	FDR P	pη^2^
VL	Pre-intervention	0.52 ± 0.16	0.49 ± 0.09	​	​	​
	Post-intervention	0.49 ± 0.12	0.47 ± 0.13	​	​	​
	Group main effect	​	​	0.164	0.815	0.002
	Time main effect	​	​	1.186	0.356	0.052
	Interactive effect	​	​	0.113	0.896	0.028
RF	Pre-intervention	0.29 ± 0.11	0.32 ± 0.07	​	​	​
	Post-intervention	0.29 ± 0.16	0.29 ± 0.16	​	​	​
	Group main effect	​	​	1.321	0.335	0.116
	Time main effect	​	​	0.251	0.744	0.023
	Interactive effect	​	​	0.547	0.435	0.062
VM	Pre-intervention	0.45 ± 0.12	0.43 ± 0.10	​	​	​
	Post-intervention	0.43 ± 0.11	0.44 ± 0.12	​	​	​
	Group main effect	​	​	0.067	0.862	0.002
	Time main effect	​	​	1.191	0.430	0.081
	Interactive effect	​	​	0.115	0.910	0.008
ST/SM	Pre-intervention	0.13 ± 0.06	0.13 ± 0.03	​	​	​
	Post-intervention	0.12 ± 0.05	0.17 ± 0.03	​	​	​
	Group main effect	​	​	3.764	0.072	0.271
	Time main effect	​	​	2.438	0.072	0.275
	Interactive effect	​	​	0.115	0.908	0.006
BF	Pre-intervention	0.14 ± 0.03	0.14 ± 0.03	​	​	​
	Post-intervention	0.13 ± 0.04	0.16 ± 0.04	​	​	​
	Group main effect	​	​	5.153	0.049	0.213
	Time main effect	​	​	4.523	0.071	0.178
	Interactive effect	​	​	3.130	0.049	0.163

VL, means Vastus Lateralis; RF, means Rectus Femoris; VM, means Vastus Medialis; ST/SM, means Semitendinosus/Semimembranosus; BF, means Biceps Femoris.

#### 3.3.4 Co-activation ratio

The Shapiro-Wilk test indicated that the co-activation ratio data for the flexor-extensor muscles during the landing absorption phase followed a normal distribution.

The repeated measures ANOVA of the knee flexor–extensor co-activation ratio revealed a significant main effect of group (F = 9.983, FDR-adjusted p = 0.011, pη^2^ = 0.270) and time × group interaction effect (F = 4.196, FDR-adjusted p = 0.048, pη^2^ = 0.237), while the main effect of time was not significant (F = 1.404, FDR-adjusted p = 0.362, pη^2^ = 0.094). Simple effect analysis indicated that, after the intervention, the co-activation ratio of the flexor–extensor muscles in PAP group was significantly higher than both pre-intervention (p = 0.028, 95% CI [0.009, 0.094]) and the control group (p = 0.041, 95% CI [0.001, 0.095]).

**FIGURE 8:**
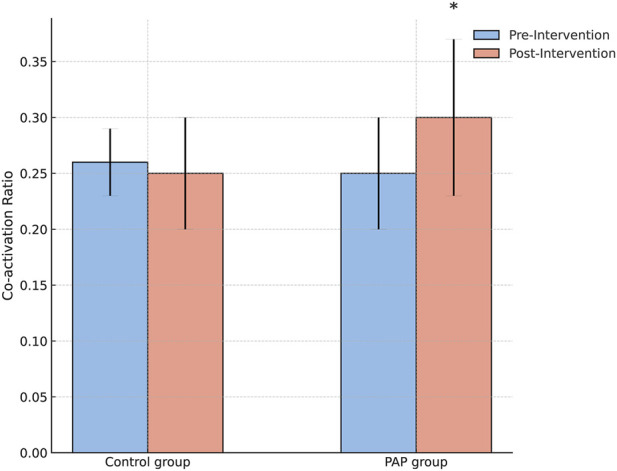
Comparison of Co-activation ratios pre- and post-intervention.

The detailed results are shown in Table 9 and Figure 8.

**TABLE 9:** Co-activation ratio of knee flexor-extensor muscles.

Time	Control group	PAP group	ANOVA		
			F	FDR P	pη^2^
Pre-intervention	0.26 ± 0.03	0.25 ± 0.05	​	​	​
Post-intervention	0.25 ± 0.05	0.30 ± 0.07	​	​	​
Group main effect	​	​	9.983	0.011	0.270
Time main effect	​	​	1.404	0.364	0.094
Interactive effect	​	​	4.196	0.048	0.237

## 4 Discussion

This study investigated the effects of post-activation potentiation (PAP) of the hamstrings on surface electromyography (sEMG) signals and biomechanical characteristics of the knee joint during landing in collegiate athletes. The primary pre-specified outcomes confirmed that hamstring PAP increased EMG power in the 60–200 Hz band for both the biceps femoris and semitendinosus/semimembranosus. Secondary and exploratory analyses suggested additional biomechanical adaptations.

The analysis of surface electromyography (sEMG) signals demonstrated that the conditioning contraction significantly altered hamstring activation patterns during landing, primarily reflected by an overall increase in EMG signal amplitude. Specifically, following the Maximum Voluntary Isometric Contraction (MVIC) intervention, the integrated EMG (iEMG) of the hamstrings during landing was significantly higher than that observed at baseline, indicating an elevation in hamstring activation magnitude after the intervention. These changes likely reflect alterations in voluntary neuromuscular activation patterns associated with activation history during a dynamic landing task. These findings are consistent with previous observations that voluntary EMG amplitude may increase following a conditioning contraction (Sale, 2002; Scott et al., 2018).

Further analysis of the wavelet-decomposed EMG signals revealed distinct and muscle-specific responses of the semitendinosus/semimembranosus and biceps femoris to the PAP intervention. In the ST/SM, post-activation potentiation primarily increased power in the mid-to-high frequency bands (60–200 Hz and 200–480 Hz), while changes in the low-frequency range (0–60 Hz) were minimal. These findings indicate a frequency-dependent redistribution of surface EMG power in the medial hamstrings during landing after the intervention.

Functionally, the semitendinosus and semimembranosus (ST/SM) contribute to knee joint stabilization during landing by assisting in the control of tibial rotation and frontal-plane motion. Accordingly, the observed increase in higher-frequency surface EMG power may indicate a task-related change in the frequency-domain characteristics of the ST/SM signal during landing. In contrast, the biceps femoris (BF) exhibited a broader and less localized redistribution of EMG power across frequency bands. In contrast, the biceps femoris showed a significant increase in the primary 60–200 Hz band and an exploratory increase in the 200–480 Hz band, whereas the 0–20 Hz band showed a significant decrease. This pattern suggests a broader redistribution of BF surface EMG power across frequency bands after the conditioning contraction, rather than a uniform increase across the spectrum.

Although previous studies have reported differences in fiber-type composition between the biceps femoris and the medial hamstrings (Schiaffino, 2010), the present study did not assess muscle fiber characteristics or intrinsic contractile properties. Therefore, changes in EMG spectral content cannot be directly attributed to fiber-type–specific mechanisms, calcium dynamics, or myosin regulatory light chain phosphorylation.

From a mechanistic perspective, PAP has traditionally been associated with changes in excitation–contraction coupling, myosin regulatory light chain phosphorylation, and alterations in contractile responsiveness, particularly in fast-twitch muscle fibers (Moore and Stull, 1984; Sale, 2002; Allen et al., 2008; Blazevich and Babault, 2019). These mechanisms may provide a theoretical basis for understanding why a prior maximal contraction can acutely modify subsequent muscle activation and performance. However, the present study did not include controlled electrical stimulation, single-motor-unit recordings, muscle fiber assessment, or direct measures of neural drive. Therefore, the observed frequency-domain changes in surface EMG should not be interpreted as direct evidence of specific motor unit recruitment strategies, fiber-type–specific activation, or cellular potentiation mechanisms.

A more conservative interpretation is that the hamstring conditioning contraction was associated with muscle-specific redistribution of surface EMG power during landing. This redistribution may reflect the combined influence of activation history, landing mechanics, muscle–tendon behavior, electrode and tissue-filtering effects, and signal-processing characteristics. In the present study, the medial hamstrings showed a relatively more confined increase in higher-frequency components, whereas the biceps femoris demonstrated a broader redistribution across frequency bands. This heterogeneity suggests that different hamstring components may respond differently to a conditioning contraction during rapid landing, which may be related to their distinct functional roles in dynamic knee joint control(Bocheng et al., 2024). Nevertheless, these findings should be regarded as changes in recorded surface EMG characteristics rather than direct evidence of underlying neural or cellular mechanisms. Future studies incorporating high-density EMG, motor-unit decomposition, twitch interpolation, or muscle-specific imaging may help clarify the physiological sources of these frequency-domain responses.

The findings of this research demonstrate that hamstring PAP was associated with acute changes in the biomechanical characteristics of the knee joint in the sagittal plane during landing. Compared to pre-intervention, participants exhibited increased knee flexion angles and reduced extension moments during landing. These changes may contribute to reducing the risk of non-contact ACL injuries (Brown et al., 2014). Previous studies have shown that when an anterior force is applied to the tibia, causing it to translate forward, the degree of anterior tibial displacement tends to decrease as the knee flexion angle increases (Engebretsen et al., 2012). Conversely, at smaller knee flexion angles, the quadriceps have a greater lever arm to exert anterior pulling forces on the tibia, resulting in higher anterior shear forces at the knee joint (DeMorat et al., 2004). Numerous studies have previously demonstrated that strengthening the hamstrings through training improves the hamstring-to-quadriceps strength ratio, indicating that increased hamstring strength can effectively counteract the quadriceps’ pull on the tibia, thereby reducing the risk of ACL injuries (Serpell et al., 2015; Heinert et al., 2021; Kellis et al., 2023). The results of this study suggest that the acute increase in hamstring strength induced by PAP may produce a protective effect similar to that achieved through training, within the duration of the PAP effect.

The PAP of the hamstrings also led to changes in the biomechanical characteristics of the knee joint in the coronal plane. Specifically, PAP significantly reduced knee valgus angles during landing and decreased abduction moments at the point of maximum knee flexion. This change is considered a more favorable landing strategy. Numerous studies have linked larger valgus angles and abduction moments during landing to a higher risk of ACL injury. For instance, Hewett et al. (2005) demonstrated that increased knee abduction moments during landing were predictive of ACL injury in female athletes. Similarly, Krosshaug et al. (2007) conducted a video analysis of 39 ACL injuries in basketball and found that a combination of knee valgus and internal rotation often accompanied ACL rupture, suggesting that excessive frontal-plane loading plays a critical role in injury mechanisms.

However, we also observed no statistically significant difference in knee abduction moments within 40° of knee flexion or at maximum abduction moment compared to pre-activation. This suggests that PAP of the hamstrings may not substantially reduce kinetic risk factors for ACL injury originating from the coronal plane. Previous studies have indicated that as knee flexion angles increase, the role of the ACL in resisting anterior tibial translation diminishes (Dauty et al., 2022), suggesting that the hamstring PAP may not alleviate coronal plane loads during the period when ACL stress is greatest. Furthermore, recent research has challenged the previously emphasized importance of knee abduction moments in ACL rupture risk (Boden et al., 2009; Mokhtarzadeh et al., 2013; Bakker et al., 2016; Carlson et al., 2016), suggesting that changes in coronal plane biomechanics (valgus angles and abduction moments) may not be primary risk factors for ACL injury (Boden and Sheehan, 2022). Thus, the relationship between coronal plane biomechanical characteristics and ACL injury risk remains a topic of ongoing debate.

Biomechanical changes in the horizontal plane are also of particular interest in this study. Previous research has demonstrated that dynamic changes in the transverse plane are closely linked to ACL loading. For example, the study by Fleming et al. (2001) showed that internal rotation moments at the knee significantly increase ACL stress under weight-bearing conditions. In our study, while hamstring PAP did not alter the kinematic characteristics of the knee during landing, it did reduce internal rotation moments, which suggests a potential reduction in the risk of ACL injury. One explanation for how hamstring activation might influence the biomechanical characteristics of the knee in the transverse plane is selective activation of the medial and lateral hamstrings to control tibial rotation (Ciccotti et al., 1994). Some studies, such as that by Besier et al. (2003), suggest that during dynamic landing, the hamstrings are pre-activated, with a tendency to preferentially activate the biceps femoris (the lateral hamstring) to counteract internal rotation moments. Additionally, the physiological mechanism of PAP, now widely believed to involve enhanced phosphorylation of myosin regulatory light chains and increased post-activation neural activity, leading to greater motor unit recruitment, might also play a role (Blazevich and Babault, 2019). Considering this, we hypothesize that PAP may have increased participants’ muscular strength and neuromuscular control prior to and during the early stages of landing. This enhanced capacity likely allowed them to more effectively counteract internal rotation moments at the knee during dynamic landing tasks, thereby potentially reducing the risk of ACL injury.

Current research evidence suggests that ACL loading may not originate from a single plane but rather from a combination of multi-planar forces (Markolf et al., 1995; Oh et al., 2012). For instance, video analyses of numerous ACL injuries have shown that at the moment of injury, the knee joint often exhibits a combination of a small flexion angle, a large valgus angle, and significant internal rotation (Olsen et al., 2004; Shimokochi and Shultz, 2008). Additionally, a combined loading pattern of internal rotation, extension, and abduction moments at the knee is also believed to place maximum strain on the ACL (Markolf et al., 1995; Oh et al., 2012). These combined biomechanical characteristics collectively contribute to the occurrence of ACL injuries. Moreover, as the knee flexion angle increases, the load borne by the ACL decreases (Dai et al., 2019), with peak shear forces on the ACL typically occurring at an average knee flexion angle of 37° (DeMorat et al., 2004), which coincides with the first peak of the vertical ground reaction force. Therefore, it is essential to examine the multi-planar joint moments within the knee flexion angle range associated with the highest injury risk.

In this study, participants demonstrated a significant reduction in both maximum extension moment and internal rotation moment within 40° of knee flexion during the landing task, while no significant differences were observed in abduction moments compared to pre-activation. During the landing task, the knee transitions from an open kinetic chain before foot contact to a closed kinetic chain upon ground contact. Throughout this process, the knee’s multi-planar kinematic characteristics undergo dynamic adjustments to maintain joint stability. The role of the hamstrings in this phase is to work synergistically with the quadriceps to limit anterior tibial translation (Carlson et al., 2016), while also controlling knee rotation when dynamic valgus occurs and the joint transitions into a closed kinetic chain (Hewett et al., 2016), thus reducing the load on the ACL.

The biomechanical patterns observed after hamstring PAP in the present study show some similarities to movement adaptations reported in injury prevention and neuromuscular training programs. For example, plyometric training has been shown to increase knee flexion angles and reduce valgus angles during landing (Myer et al., 2006), while core stability training has been associated with increased knee flexion angles and reduced internal rotation angles in female athletes (Pfile et al., 2013). These similarities suggest that acute hamstring-focused conditioning may influence certain landing-related biomechanical characteristics in a direction that overlaps with selected outcomes previously reported after neuromuscular training, although the mechanisms and practical implications may differ substantially. However, this interpretation should be made cautiously because the present study examined an acute laboratory-based conditioning protocol rath er than a long-term field-based injury prevention program. In sports such as basketball, performing a prone MVIC exactly 8 min before a landing task is unlikely to be practical. Therefore, the present protocol should be interpreted primarily as a mechanistic model demonstrating that brief hamstring-focused conditioning can acutely modify hamstring EMG characteristics and knee joint biomechanics during landing. From a field-translation perspective, similar neuromuscular preparation may be more feasible if incorporated into pre-training or pre-competition warm-up routines using practical hamstring activation exercises, such as resisted knee flexion, Nordic hamstring variations, bridge-based isometric contractions, or flywheel hamstring exercises. Future studies should determine whether these field-based alternatives can induce comparable changes in landing-related neuromuscular and biomechanical outcomes under sport-specific conditions.

## 5 Limitations

Several limitations should be acknowledged. First, the relatively small sample size and single-center design may limit statistical power and generalizability. Second, only male university athletes were included, restricting the applicability of the findings to female athletes, non-athletic individuals, and other age groups. Given the higher risk of non-contact ACL injury in female athletes and potential sex-related differences in neuromuscular control and landing mechanics, future studies should specifically examine whether this hamstring conditioning protocol produces comparable EMG and biomechanical effects in female athletes. Third, the present study used surface EMG only. Although wavelet decomposition provides information on the time-frequency characteristics of the EMG signal, surface EMG cannot directly identify individual motor unit behavior, neural drive, muscle fiber-type recruitment, or cellular potentiation mechanisms. Therefore, the observed frequency-domain changes should be interpreted as alterations in surface EMG characteristics rather than direct evidence of underlying neural mechanisms. Finally, the outcomes were assessed only acutely during a controlled laboratory-based 50 cm drop-landing task. The relatively large knee flexion angle may reflect a standardized soft-landing strategy and should not be directly generalized to sport-specific landings. Although FDR correction was applied, the large number of biomechanical and EMG comparisons still warrants cautious interpretation. Larger multicenter studies including female athletes, crossover designs, sport-specific tasks, and longer follow-up are needed to confirm the external validity and functional relevance of these findings.

## Data Availability

The raw data supporting the conclusions of this article will be made available by the authors, without undue reservation.
